# Supplementary material for: Learning curve and surgical outcome of robotic assisted colorectal surgery with ERAS program
Source: Sci Rep. 2022 Nov 29;12:20566. doi: 10.1038/s41598-022-24665-w (PMC9709162; doi:10.1038/s41598-022-24665-w)
Supplement: Supplementary file 1 — Supplementary Information 1. [file 41598_2022_24665_MOESM1_ESM.zip › LSC Raw data-Ñ[▒K/120/O_ñw╝╨░O▒Kñσ.pdf]

正式摘要(電子簽章：0000002122237028)

請選擇...reporter

臺中榮民總醫院

Operation Note

[redacted]

- 手術日期：2021/03/12
- 手術醫師：VS 蔣鋒帆，CR 林陳彥，Fellow 黃俋霖，R 劉佳樺
- Anesthesia : GE
- Position : Lithotomy
- Aseptic agents : Betadine Aqua , Zephnan

Diagnosis

- Pre-operative diagnosis : Sigmoid colon cancer
- Post-operative diagnosis : s/p laparoscopic low anterior resection

Surgical Indication

- For removing the primary tumor

Operation

- 1. Laparoscopic low anterior resection was done
  2. Intraoperative colonoscopy : (-)
  3. High ligation (+) : At IMA root. IMV and left colic artery were also ligated.
  4. EndoGIA COVIDIEN ENDO GIA 60mm (Medium Thick) X2 + EEA (29 mm) were applied.
  5. Proximal cut end: 15 cm, distal cut end: 2 cm
  6. The colon was not taken down from the splenic flexure site
  7. ADHESION BARRIER FILM was placed below the central abdominal wound.

Finding

- 1. A 4 x 4 cm, pedunculated tumor. The stalk base was at distal S-colon area, but the main tumor would drop to distal rectal site.
  2. Some ascites was presented over the pelvic area.
  3. There was no obvious visible liver metastasis, no visible peritoneal seeding.
  4. Bilateral doughnuts : intact ; Air leak test: Done and no air leak was found

Procedure

- 1. General endotracheal anesthesia was induced and then the patient was placed in modified Trendelenburg lithotomy position.
  Preoperative antibiotics were given. A Foley catheter and a nasogastric tube were placed. The abdomen was prepped and draped in standard sterile fashion.
  2. A small vertical incision was made right next to the umbilicus and a 12 mm trocar was inserted into the peritoneal cavity by open method. Peumoperitoneum was established with CO2 inflation. Laparoscopy was inserted and then another 12 mm trocar was inserted over RLQ. Extra three 5 mm trochars were inserted over RUQ, LUQ and LLQ of abdomen. The operative findings were mentioned as above. Adhesion lysis was performed via Harmonic scalpel.
  3. The small bowel was inspected and retracted to the right by grasper. The sigmoid colon was then held by
  grasper and the peritoneum overlying the mesentery was then scored with electrocautery. The inferior mesentery
  artery was identified, ligated below the left colic artery with endoclips, and transected, so was inferior

  mesentery vein. Dissection was done above the Gerota's fascia and toward the peritoneal attachments.

- 4. The arcade vessels to the sigmoid colon were also divided. Distal descending colon and upper rectum were mobilized.
  5. The colon was mobilized from retroperitoneum overlying the sacral promontory and into the pelvis. The incision was extended laterally and medially across the peritoneal reflection.
  6. The mesorectum was then mobilized using sharp dissection over the level of pelvic floor muscle. Then distal descending colon and rectum were mobilized as possible.
  7. One staple of EndoGIA COVIDIEN, ENDO GIA 60mm (Medium Thick) was applied to divide the upper rectum site.
  8. Incision was further extended from the middle umbilicus trochar site. The proximal colon was then taken out for carefully inspection, and to make sure it has an excellent blood supply.
  9. The field was isolated with moist pad and the bowel opened and sized. It was determined that a 29 mm circular stapler could be accommodated. A pursestring suture of 2-0 prolene was placed, the anvil was inserted, and then the pursestring suture was tied.
  10. However, further specimen revealed that the lower edge of tumor was transected by the staple. (See picture for more information)
  11. Therefore, another staple of EndoGIA COVIDIEN, ENDO GIA 60mm (Medium Thick) was applied over the distal rectum area for adequate safe margin. The specimen was removed.
  12. DRE was performed to check for no residule tumor.
  13. The circular stapler was introduced through the anus and pierced through the mid-portion of the stapler line.
  14. The proximal bowel with anvil in place was engaged with the circular stapler and the stapler was then fired. Two complete donuts were obtained and the distal one was submitted to pathology for margin confirmation.
  15. The anastomosis was tested for leaks with irrigation solution and air introduced through the rectum and was found to be patent and intact.
  16. The possible bleeder was then checked thoroughly.
  17. The ADHESION BARRIER FILM was then placed below the umbilical wound.
  18. The fascia of midline wound was closed with interrupted sutures via Num. 1 Vicryl.
  19. After irrigation the wound with N/S, the skin was closed with interrupted 3-0 nylon.
  20. The patient tolerated the procedure well and was brought to the POR in stable condition
- Estimated blood loss: minimum
- 器械數量: 正確
- 紗布數量: 正確

Image

- PAINT
  - abdomen

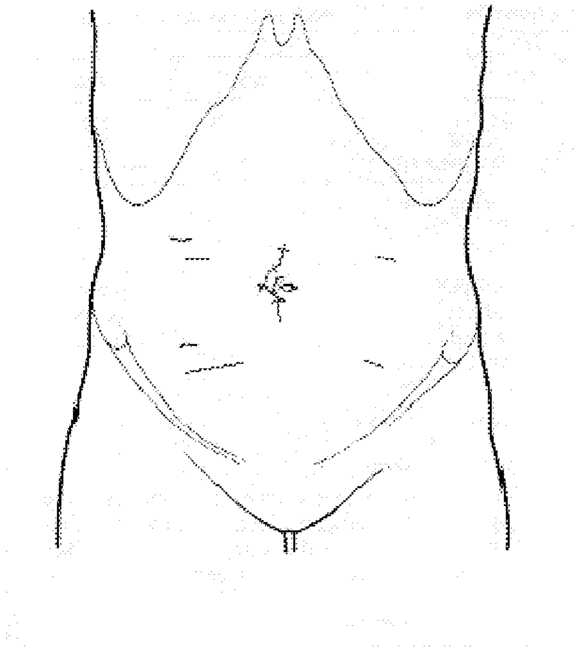

  - colon & rectum

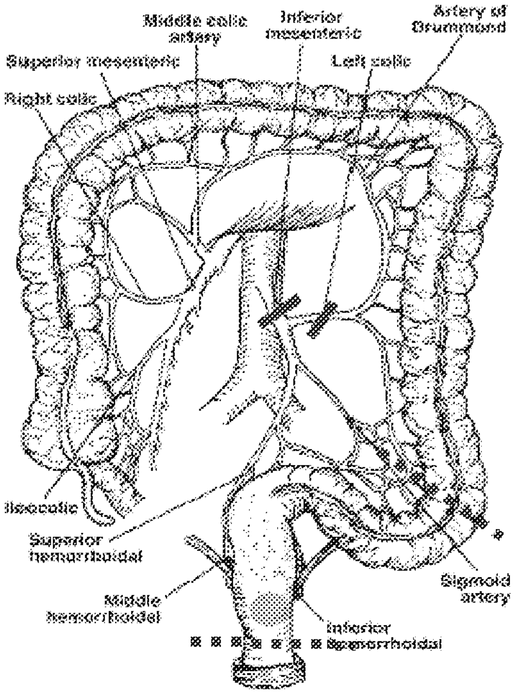

- The resected rectum

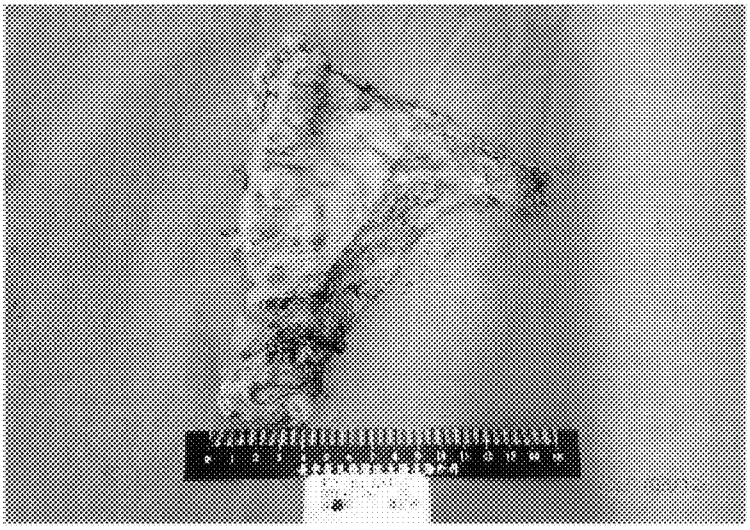

The stalk base was marked as below. The tumor dropped to distal rectal site

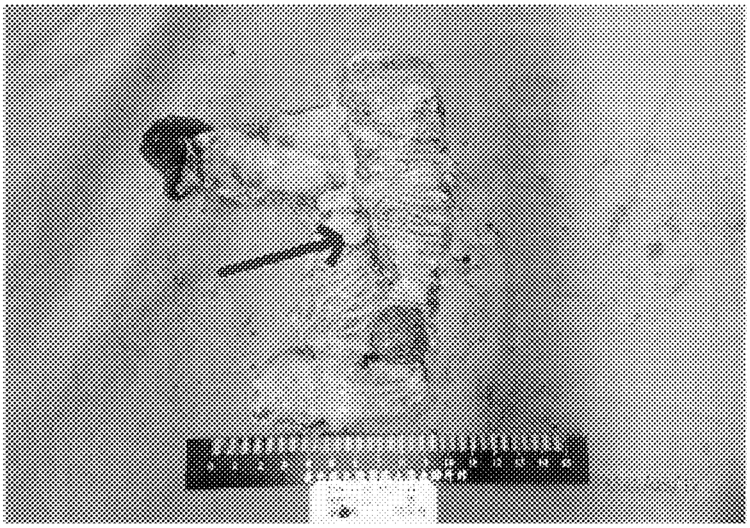

Initially, the tumor was transected by staple. Therefore, another resection was done again for adequate margin.

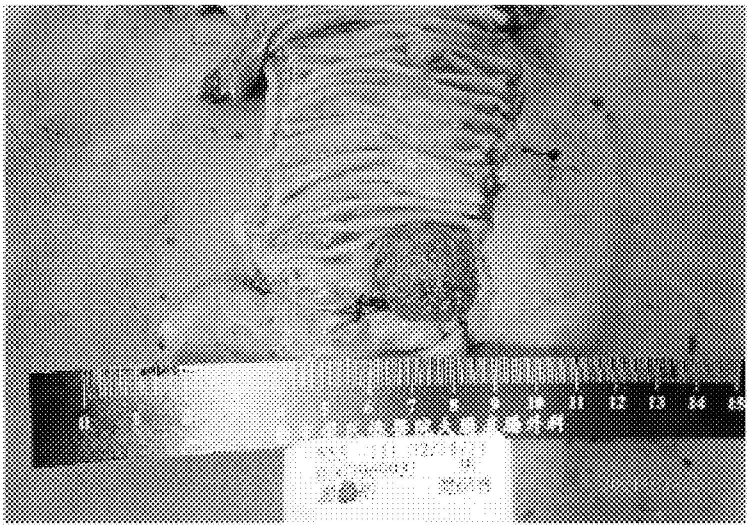

主任 2062F蔣鋒帆 / 醫師 2301H林陳彥

正式摘要(電子簽章：**000000212237028)**

請選擇...reporter

doctorRank:B
